# Selective resistance profiles emerging in patient-derived clinical isolates with cabotegravir, bictegravir, dolutegravir, and elvitegravir

**DOI:** 10.1186/s12977-018-0440-3

**Published:** 2018-08-17

**Authors:** Maureen Oliveira, Ruxandra-Ilinca Ibanescu, Kaitlin Anstett, Thibault Mésplède, Jean-Pierre Routy, Marjorie A. Robbins, Bluma G. Brenner, the Montreal Primary HIV (PHI) Cohort Study Group, Mario Legault, Jean-Guy Baril, Louise Charest, Marc-André Charron, Pierre Côté, Alexandra de Pokomandy, Serge Dufresne, Claude Fortin, Jason Friedman, Norbert Gilmore, Emmanuelle Huchet, Marina Klein, Louise Labreque, Richard Lalonde, Roger Leblanc, Bernard Lessard, Catherine Milne, Marie Munoz, Martin Potter, Danielle Rouleau, Jean-Pierre Routy, Jason Szabo, Réjean Thomas, Cecile Tremblay, Benoît Trottier, Sylvie Vézina

**Affiliations:** 1 McGill University AIDS Centre, Lady Davis Institute for Medical Research, Jewish General Hospital, 3755 Côte Ste-Catherine Road, Montreal, QC H3T 1E2 Canada; 2 Department of Microbiology and Immunology, McGill University, Montreal, QC Canada; 3 Faculty of Medicine (Surgery, Experimental Medicine, Infectious Disease), McGill University, Montreal, QC Canada; 4 BC Children’s Hospital Research Institute, Vancouver, BC Canada

**Keywords:** HIV-1, Integrase inhibitors, Antiretroviral drug resistance, Cell culture selections, Primary HIV infection isolates, HIV subtypes

## Abstract

**Background:**

Integrase strand transfer inhibitors (INSTIs) are recommended for first-line HIV therapy based on their relatively high genetic barrier to resistance. Although raltegravir (RAL) and elvitegravir (EVG) resistance profiles are well-characterized, resistance patterns for dolutegravir (DTG), bictegravir (BIC), and cabotegravir (CAB) remain largely unknown. Here, in vitro drug selections compared the development of resistance to DTG, BIC, CAB, EVG and RAL using clinical isolates from treatment-naïve primary HIV infection (PHI) cohort participants (n = 12), and pNL4.3 recombinant strains encoding patient-derived Integrase with (n = 5) and without (n = 5) the E157Q substitution.

**Results:**

Patient-derived viral isolates were serially passaged in PHA-stimulated cord blood mononuclear cells in the presence of escalating concentrations of INSTIs over the course of 36–46 weeks. Drug resistance arose more rapidly in primary clinical isolates with EVG (12/12), followed by CAB (8/12), DTG (8/12) and BIC (6/12). For pNL4.3 recombinant strains encoding patient-derived integrase, the comparative genetic barrier to resistance was RAL > EVG > CAB > DTG and BIC. The E157Q substitution in integrase delayed the advent of resistance to INSTIs. With EVG, T66I/A, E92G/V/Q, T97A or R263K (n = 16, 3, 2 and 1, respectively) arose by weeks 8–16, followed by 1–4 accessory mutations, conferring high-level resistance (> 100-fold) by week 36. With DTG and BIC, solitary R263K (n = 27), S153F/Y (n = 7) H51Y (n = 2), Q146 R (n = 3) or S147G (n = 1) mutations conferred low-level (< 3-fold) resistance at weeks 36–46. Similarly, most CAB selections (n = 18) resulted in R263K, S153Y, S147G, H51Y, or Q146L solitary mutations. However, three CAB selections resulted in Q148R/K followed by secondary mutations conferring high-level cross-resistance to all INSTIs. EVG-resistant viruses (T66I/R263K, T66I/E157Q/R263K, and S153A/R263K) retained residual susceptibility when switched to DTG, BIC or CAB, losing T66I by week 27. Two EVG-resistant variants developed resistance to DTG, BIC and CAB through the additional acquisition of E138A/Q148R and S230N, respectively. One EVG-resistant variant (T66I) acquired L74M/G140S/S147G, L74M/E138K/S147G and H51Y with DTG CAB and BIC, respectively.

**Conclusions:**

Second generation INSTIs show a higher genetic barrier to resistance than EVG and RAL. The potency of CAB was lower than BIC and DTG. The development of Q148R/K with CAB can result in high-level cross-resistance to all INSTIs.

## Background

Over the past 40 years, remarkable advances in antiretroviral therapy has enabled people living with HIV to enjoy longer life expectancy and an improved quality of life. Despite these advances, ongoing development of more robust and durable drug regimens remain critical to avoid the long-term risk of drug resistance and treatment failure [1, 2]. Presently, integrase strand transfer inhibitors (INSTIs) are the favored class of drugs in first-line combination therapy based on their high potency, improved tolerability, low toxicity and high genetic barrier to resistance [3–5].

Raltegravir (RAL) and elvitegravir (EVG) were the first INSTIs to be approved in 2007 and 2012, respectively [6, 7]. Although highly efficacious in the management of HIV, both RAL and EVG were shown to be prone to the development of resistance when used in salvage therapy without other active antiretroviral drugs [8, 9]. The resistance and cross-resistance profile for RAL and EVG have been well described [8, 10, 11]. Drug resistance is associated with the accumulation of primary resistance substitutions and relevant compensatory substitutions along several pathways including the (1) N155H and G140A/G148R/H/Q pathways conferring high level cross-resistance to RAL and EVG; (2) the T66I or E92Q/G pathways leading to resistance to EVG; or (3) the Y143R/H/C RAL-specific resistance pathway [4, 12].

The emergence of viruses displaying resistance and cross-resistance to RAL and EVG spurred research into the development of “second-generation” INSTIs [4, 12]. These include dolutegravir (DTG) that was approved in 2013, bictegravir (BIC) that was approved in the US in February 2018 and cabotegravir (CAB) that is in phase III clinical development with anticipated release in 2019 [13–15]. Dolutegravir displays a higher genetic barrier to resistance than RAL and EVG, retaining efficacy against many RAL- and EVG-resistant variants [16–18]. In the SAILING clinical study, only 4 of 354 treatment-experienced, INSTI-naïve patients treated with dolutegravir acquired integrase-inhibitor resistance substitutions upon virological failure. Two of these individuals acquired the R263K mutation [19] that had previously been selected in culture by DTG to confer low-level resistance [20]. Interestingly, R263K-containing viruses do not seem able to acquire compensatory substitutions that might increase levels of resistance [21–24]. In cell culture selections with DTG, the emergence of singleton R263K, S153Y or H51Y mutations confers low-level resistance (< 3-fold resistance) and contributes to a significant negative impact on viral replicative fitness [4, 20, 25, 26]. DTG has been shown to have a longer binding half-life to the HIV integrase enzyme than either RAL or EVG which may help to explain why it maintains activity against most RAL or EVG resistant variants [27, 28].

The recent phase 3 randomized GS-US-380-1489 and GS-US-380-1490 clinical trials demonstrated bictegravir BIC, co-formulated with tenofovir alafenamide and emtricitabine, to be non-inferior to DTG, when co-formulated with either abacavir and lamivudine or tenofovir alafenamide and emtricitabine at week 48 [13, 15, 29]. No resistance to any drug was observed with either BIC- or DTG-based regimens. BIC and DTG showed high barriers to in vitro resistance in MT-2 cells, with emergent M50I and R263K conferring low-fold resistance in the nM range [30]. BIC, like DTG, appears to show broad-range potency against viruses with primary INSTI mutations conferring resistance to RAL or EVG [31].

CAB, a structural analogue of DTG, displays unique physicochemical and pharmacokinetic properties that permits formulation as a single oral tablet for daily dosing and as a long-acting nanosuspension for monthly to quarterly intramuscular injection. Toxicity profiles for CAB, like that reported for DTG, BIC and RAL, include headache, nausea and diarrhea, [5, 13, 32, 33]. Long-acting CAB showed mild to moderate injection-site reactions that rarely resulted in treatment discontinuation (< 1%) [14]. The ability of injectable CAB to achieve and maintain clinically relevant plasma concentrations for 16 weeks may circumvent the need for daily dosing, allowing for long-acting treatment and pre-exposure prophylaxis (PreP) regimens [14]. The LATTE-2 clinical trial showed non-inferiority of the two-drug oral CAB plus rilpivirine (RIL) regimen to the CAB-abacavir-lamivudine drug regimen, with 2/115 experiencing virological failure. One patient acquired virus with the Q148R mutation, conferring phenotypic resistance to RAL, EVG and CAB, in association with the K103N, E138G, and K238T, conferring cross-resistance to non-nucleoside RT inhibitors (NNRTIs) [14]. Similarly, at week 48 in the Phase 2 Latte study, the injectable long-acting formulation of CAB and RIL was non-inferior to efavirenz on a dual nucleoside reverse transcriptase inhibitor (NRTI) backbone with one virological failure in injectable CAB/RIL arm harbouring viruses acquiring the INSTI-associated Q148R mutation with the E138Q NNRTI-resistant mutation [32].

To gain a better understanding of the possibility of resistance to newer INSTIs, we performed in vitro cell culture selections with DTG, BIC, CAB and EVG against primary isolates from newly infected persons harbouring subtype B (n = 7) and non-B subtype (n = 5) infections. In addition, recombinant strains of HIV-1 with integrase derived from clinical isolates assessed the impact of E157Q substitution on emergent resistance to relevant INSTIs was assessed at weeks 8, 16, 24 and 46. We observed a higher genetic barrier to DTG and BIC as compared to CAB across the different viral subtypes.

## Results

### Differential selection of resistance to newer integrase inhibitors

This study was designed to compare the differential ability of a panel of patient-derived clinical isolates (n = 12) and recombinant strains (n = 10) to develop resistance to the integrase inhibitors, DTG, BIC, CAB and EVG. Baseline integrase natural polymorphisms, viral subtype and GenBank accession numbers are summarized in Tables 1 and 2.

**Table 1:** Baseline natural integrase polymorphisms for the HIV-1 clinical isolates used for the in vitro selections with integrase inhibitors

Isolate ID	GenBank accession number	Subtype	Natural polymorphisms in integrase
14514	KT988124	B	K7R, D10E, S17N, M50I, K111Q, T112V, G123S, T125V, R127K, I220L, Y227F, N232D, D256E
10387	KX7140173	B	D10E, V31I, L45Q, K111E, I113V, G123S, A124T, T125A, R127K, M154I, V201I, D207N, N232D, L234I
10249	KX714014	B	D10E, V31I, L45Q, L101I, K111E, I113V, G123S, A124T, R127K, D207N, N232D, L234I
14624	KX714018	B	D10E, S17N, A23V, L28I, S39C, V72I, L101I, G123S, R127K, N232D
14637	KT988125	B	D10E, E11D, R20K, V31I, S39N, M50I, V72I, S119T, G123S, A124N, R127K, G193E, V201I, D286N
14947	KT988126	B	D10E, E11D, R20K, V31I, S39N, M50I, S119T, G123S, A124N, R127K, G193E, V201I, T218S, D286N
5326	KX714021	B	K7R, D10E, E11D, K14R, V31I, V32I, M50L, V72I, L101I, G123S, A124N, R127K, S195T, I203M, I220L, Y227F, N232D
4742	MG805951	C	D10E, V31I, S39C, V72I, I84M, Q95P, F100Y, L101I, T112V, G123S, A124N, T125A, R127K, D167E, V201I, I203M, K215N, T218L, N232D, L234I, D278A, S283G, R284G
10947	MG805955	C	D6E, D25E, V31I, L45Q, M50I, V72I, P90S, T93S, F100Y, L101I, G106A, T112V, G123S, T125A, R127K, K136Q, V201I, T218I, N232D, L234I, R269K, D278A, S283G, D288N
6343	MG805950	CRF01_AE	D10E, K14R, A21T, V31I, S39N, T112V, G123S, T125A, R127K, G134N, I135V, K136R, D167E, V201I, N232D, L234I, S283G
14515	MG805952	CRF02_AG	D10E, E11D, R20K, A21T, V31I, V72I, L101I, T112V, G123S, T125A, R127K, G134N, K136Q, D167E, V201I, N232D, L234I, V249I, S283G
96USSN20	MG805953	AG	D10E, K14R, V31I, V72I, L101I, T112V, G123S, T125A, R127K, G134N, I135V, K136T, V201I, T206S, N232D, L234I, D256E, R269K, S283G
pNL4.3		B	D10E, I113V, S119R, G123S, A124T, R127K, V151I, L234V

**Table 2:** Treatment status and baseline natural integrase polymorphisms of the HIV-1 recombinant viruses used for the in vitro selections with integrase inhibitors

Sample ID	GenBank accession number	Treatment status	Integrase baseline polymorphisms
E78001	MH513660	ART-naïve	D10E, V31I, L68LV, V72I, I73IV, T112IT, I113V, G123S, R127K, I162IV, V201I, N232D, R284GR
E78003	MHS13661	ART-naive	D10E, E11D, S24N, V31IV, V32I, L45IL, V72I, L101I, T112A, S119PRST, T122IT, G123S, A124AT, R127K, K136KN, V201IV, N232D
E78004	MHS13662	ART-naïve	D10AE, E11D, V37I, K111A, T112A, S119P, G123S, A124T, T125A, R127K, V201IV, T206S, I208IL, N232D
E78005	MHS13663	ART-naive	D10E, V72I, L101I, G123S, A124T, R127K, I203M, T206ST, N232D
E78060	MHS13664	ART-naive	L45V, V72I, L74I, L101I, S119G, G123S, A124T, R127K, A128AT, N232D
E78110	MHS13665	ART-naive	D10E, E11D, V31I, A91AT, L101I, S119T, G123S, A124T, R127K, K156N, *E157Q*, F181L, V201I, K211R, N232D, A265AV, I268IL
E102430^†^ (subtype D)	MHS13666	INSTI (DTG)-experienced	D10E, S17N, K34KR, L45I, V72I, L101I, T112V, G123S, T125A, R127K, G134DG, I141IV, *E157Q*, K160E, D167E, G193E, V201I, I220V, N232D, L234I, D270DN, D288N
E102952^†^ (subtype D)	MHS13667	INSTI (DTG)-experienced	D10E, S17N, K34KR, L45I, V72I, L101I, T112IV, G123S, T125A, R127K, *E157Q*, K160E, D167E, G193E, V201I, I220V, N232D, L234I, D288N
E103211*	MHS13668	INSTI-naive	D10E, S17N, V31I, V72I, L101I, K111R, T112A, S119RS, T122IT, G123CS, A124N, R127K, *E157Q*, K160Q, V201I, K215N, N232D, D256E, S283GS
E103212*	MHS13669	INSTI-naive	D10E, S17N, V31I, V72I, L101I, K111R, T112A, S119RS, T122IT, G123CS, A124NT, T125AT, R127K, *E157Q*, K160Q, V201I, K215N, N232D, D256E

Italic refers to the presence of the E157Q substitution at baseline

^†^E102430 and E102952 contain integrase from the same patient

* E103211 and E103212 contain integrase from the same patient. Blood samples were drawn a few months apart for each of the patients

Primary viral isolates from PHI cohort participants harbouring subtype B (n = 7) and non-B subtype (n = 5) infections were serially passaged over the course of 46 weeks in the presence of escalating concentrations of DTG, BIC, CAB and EVG (Table 1). Tissue culture selections were performed using PHA-stimulated CBMCs since newly-infected persons typically use the CCR5 receptor and do not grow in MT-2 cells.

Cell culture selections of resistance were conducted in parallel with DTG, BIC, CAB and EVG under the same conditions. DTG, BIC and CAB exhibited high subnanomolar potency against primary HIV-1 isolates grown in CBMCs with baseline inhibitory concentrations (IC50s) of 0.25 ± 0.25 nM, 0.25 ± 0.15 nM and 0.13 ± 0.02 nM, respectively (mean ± SD, n = 10 isolates). The progress of viral outgrowth in the presence of stepwise increasing concentrations of INSTIs was monitored over the course of 46 weeks. Drug-dose escalations for individual INSTIs were based on weekly RT assays, performed in the presence and absence of drug [34]. Genotyping at weeks 8, 16, 24–27, and 36–46 ascertained the differential acquisition and accumulation of drug-associated mutations.

**Fig. 1:**
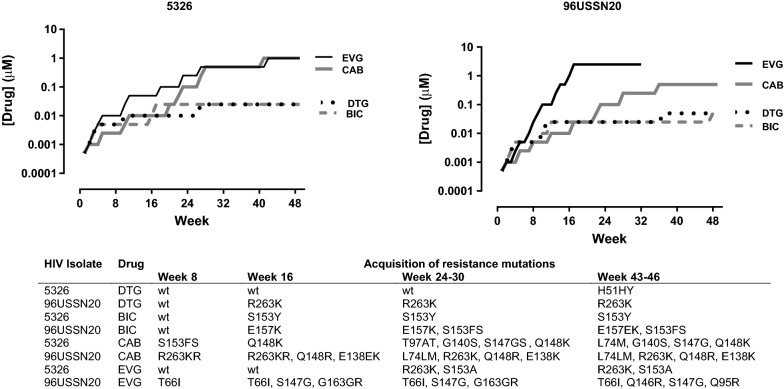
Growth of 5326 and 96USSN20 clinical isolates in escalating concentrations of dolutegravir (DTG), bictegravir (BIC), cabotegravir (CAB) and elvitegravir (EVG). The rise in drug concentrations were related to the acquisition of resistance mutations at the designated weeks of selection

The drug dose-escalations and viral outgrowth over time for HIV clinical isolates 5326 and 96USSN20 are illustrated in Fig. 1. The drug-dose escalations with DTG and BIC progressed at rates that were considerably slower than CAB or EVG. With DTG and BIC, the acquisitions of solitary mutations, including R263K, H51Y, or S153F, conferred low-level resistance to DTG or BIC. These resistance mutations conferred a negative impact on viral replication reflected in lower RT activity, precluding further escalations of DTG or BIC drug concentrations (Fig. 1).

In contrast, the respective first appearance of R263K or S153F mutations by 96USSN20 and 5326 viral strains with CAB at weeks 8, was followed by the serial accumulation of mutations along the Q148K/R resistance pathway leading to viral escape by week 48 (Fig. 1). The development of resistance to CAB progressed more slowly than EVG. For the 96USSN20 isolate, the acquisition of T66I, S147G, Q146R, and S147G conferred viral escape from EVG at week 25 (Fig. 1). Resistance codons Q146R and Q95R for this CRF002_AG isolate have been hitherto unreported. The Q146P is a reported mutation selected in vitro with EVG, reducing RAL and EVG susceptibility by 10-fold [7].The Q95K is rare nonpolymorphic accessory resistance mutation conferring little if any effect on drug susceptibility to INSTIs [7]. The acquisition of R263K with S153A by isolate 5326 conferred > 100-fold resistance to EVG.

Overall, variations in the acquisition of resistance to antiretroviral drugs are multifactorial, dependent on drug efficacy and viral heterogeneity [1]. The genetic barrier to resistance is defined by the number of mutations required to confer resistance, the level of resistance conferred by the acquired specific mutation(s) (ranging from 2 to > 1000 fold) and the resultant costs of resistance substitutions on viral replicative fitness [1]. The patterns of viral outgrowth at the final passage (week 46) for the panel of primary HIV-1 clinical isolates (n = 12) under selective pressure with DTG, BIC, CAB and EVG are shown in Table 3. Collectively, resistance selections proceeded at a considerably slower rate for DTG and BIC than CAB or EVG. By 46 weeks, resistance substitutions were observed in 12/12, 8/12, 8/12 and 7/12 of HIV isolates with EVG, CAB, DTG, or BIC, respectively (Table 3). With BIC or DTG, the acquisition of solitary resistance mutations, including R263K (n = 8), S153Y/F (n = 3) or H51H/Y (n = 1) negatively impacted on viral fitness, precluding drug-dose escalations beyond 0.005–0.025 µM.

**Table 3:** Selection of drug resistance to dolutegravir (DTG), bictegravir (BIC), cabotegravir (CAB) and elvitegravir at the final week of passage

Virus isolate	Subtype	Acquired mutations at final passage (week 46) of selective drug pressure^a^			Acquired mutations (week 26–40)^b^	
		DTG	BIC	CAB	EVG	RAL
14514	B	R263K	None	None	T66I	–
10387	B	None	None	None	T66I	E92Q
10249	B	R263K	None	None	E92Q	None
14624	B	none	None	H51HY	T66I	T97A, *N155H*
14637	B	R263K	R263K0	R263K	*T66I*, *E157Q*, *R263K*	*N155H*
14947	B	R263K	R263K	R263K, S153A	*T66I*, *E138EK*, *S147G*, *Q148R*	*Y143R*, *L74M*, *E92V*, *F121Y*, *G163GR*
5326	B	H51HY	S153Y	*L74M*, *G140S*, *S147G*, *Q148K*	*R263K*, *S153A*	*Y143R*, L74M, T97A, E157Q
4742	C	None	None	R263K	*E92EG*, *R263KR*	T66K
10947	C	R263K	R263K	S147G	*E92V*, *R263K*	–
6343	AE	R263K	S153Y	S153Y, G163R	*T66I*, *R263K*	–
14515	AG	None	R263K	None	T66I, H51HY	–
96USSN20	AG	R263K	S153FS, E157EK	*L74M*, *E138K*, *Q148R*, *R263K*	*T66I*, *Q146R*, *S147G*, *Q95R*	L74M, V79I, E138K, *G140A*, *Q148R*
-pNL4.3	B	R263K, M50I	R263K, M50I	S153F	*T66I*, *T97A*, *S147G*, *S119R*, *S153A*	*T66I*, *T97A*, *G163R*, *D232N*

Primary patient-derived viruses were passaged in CBMCs in the presence of escalating concentrations of DTG, BIC, CAB, and EVG for 46 weeks

^a^Genotypic analysis was performed of at weeks 0, 16, 24 and 46. The mutations acquired at final week of passage are listed in the order of their first appearance. Mutations highlighted in italics conferred high-level resistance. The acquired R263K, H51Y, S153Y/F mutations conferred low-level resistance with 1–10 nM final drug concentrations as compared to the high-level resistance highlighted in italics where the acquisition of complex resistance mutational motifs with CAB or EVG allowed for viral breakthrough at final drug concentrations of 0.1–2.5 µM

^b^The emerging resistance patterns to RAL determined in previous studies on viral strains are shown for comparative contextual purposes

Of note, CAB, an analogue of DTG, showed a lower genetic barrier to resistance than DTG and BIC. Although resistance patterns to CAB was like DTG and BIC for 10 of 12 isolates, two isolates showed a lower barrier to CAB resistance. Two viral variants 5326 and 96USSN20 variants acquired complex L74M/G140S/S147G/Q148K and L74M/E138K/Q148R/R263K resistant species allowing for drug-dose escalations to 1 and 0.5 µM by week 46, respectively (Fig. 1; Table 3). Two other variants, 14947 and 6343, acquired R263K/S153A and S153Y/G163R variants.

In this study, several clinical strains (14514, 10387, 10249, 14624 and 14515) showed a lower propensity to develop resistance to all tested INSTIs. Resistance profiles to DTG, BIC and CAB developed along the R263K or S153F/Y pathway; resistance to EVG was limited to the acquisition of T66I or E92Q at week 46. For the remaining seven isolates, CAB showed a lower barrier to resistance than DTG and BIC, with the acquisition of Q148R/K + 3 mutations in two isolates (Table 3).

EVG showed the lowest genetic barrier with emergent resistance observed in all 12 HIV strains (Table 3). Clinical isolates that failed to develop resistance with DTG, BIC, or CAB, including 14514, 10387, 10249, and 14515, acquired only T66I (n = 3) or E92Q (n = 1) singleton mutations with EVG at weeks 36 or 46. The other isolates accumulated 2–4 secondary drug resistance mutations along the T66I and E92QV pathways, leading to viral escape and EVG dose escalations to 1–5 µM.

Resistance was only observed in a proportion of the primary isolates. We speculate that this viral strain heterogeneity may explain why most primary viral strains rarely develop resistance to DTG and BIC in clinical settings. Our recent studies showed that select HIV-1 viral strains, including 14637 and 14947, associated with large cluster transmission outbreaks may show accelerated escape from integrase in cell culture compared with viral isolates from singleton/small clusters [25]. Large cluster strains can assist in deducing potential pathways implicated in the development of resistance to DTG, BIC and CAB (Table 3).

Different viral subtypes resulted in similar resistance profiles to INSTIs. In our previous studies, drug selections with the subtype C 4742 strain, the G118R resistance pathway arose with DTG and the Merck investigational MK2048 [35]. MK2048, the Merck investigational integrase inhibitors, showed high potency against most RAL/EVG resistant variants but its clinical development was halted due to poor pharmacokinetics [4]. The development of the G118R resistance was ascribed to a signature natural polymorphism at codon 118 in isolate 4742. In this study, 4742 developed no resistance with DTG and BIC, R263K with CAB, and E92V/R263K with EVG (Table 3).

### Resistance to integrase inhibitors using patient-derived recombinant strains

The integrase E157Q substitution has been described as a common natural polymorphism present in 2.3% of HIV-1 viral sequences, including 3.8% and 6.0% of treatment-naïve patients with subtype B and subtype CRF02_AG subtype infections, respectively (Los Alamos database, www.hiv.lanl.gov, accessed June 8, 2018). The E157Q has also been observed in several persons failing INSTI-based regimens, including RAL and DTG [36–39]. To date, very few data are available regarding virological response in patients harbouring E157Q-mutated viruses.

To assess the potential impact of E157Q on emergent resistance to INSTIs, recombinant viruses were constructed where patient-derived integrase were inserted on a pNL4-3Δ integrase background. Viruses included recombinant strains with (n = 5) and without (n = 5) the E157Q substitution (n = 5) in integrase.

**Fig. 2:**
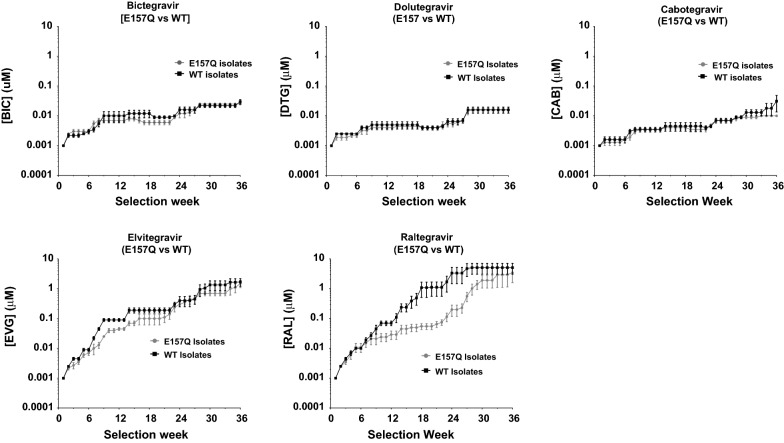
Drug dose escalations (mean ± SEM) reflect the differential emergence of resistance to integrase inhibitors by recombinant strains encoding patient-derived integrase with (n = 5) and without (n = 5) the E157Q resistance substitution

The progress of viral selections in CBMCS in stepwise increasing concentrations of DTG, BIC, CAB, EVG, and RAL over time is depicted in Fig. 2. It was noteworthy that strains harbouring the E157Q substitution showed a significantly attenuated development of resistance to RAL and EVG. The hypersensitivity of viruses to E157Q suggests that this mutation is a compensatory mutation, commonly selected with INSTIs with minimal effects on drug susceptibility [4, 40]. The E157Q mutation arose in a EVG and RAL selections in recombinant strains E78004 and E78060, respectively (Table 4).

**Table 4:** Cell culture selections of viral recombinant strains bearing the integrase from patient samples with and without the E157Q polymorphisms

Virus^a^	Codon 157	Acquired mutations at final passage (week 36–38) of selective drug pressure^b^				
		DTG	BIC	CAB	EVG	RAL
pNLWT	WT	R263K, M50I	R263K, M50I	S153F	T66I, T97A, S147G, S119R, S153A	*ND*
pNL157Q	E157Q	R263K, M50IM	Q146R	R263K, M50I	T66I, S147G, Q95K	E92Q
E78001	WT	S153F	S153Y	R263K, M50I	T66I, Q146R, S230R	*Y143R*, L74M, V151I
E78003	WT	S147G, H51Y	R263K, M50I	N155H	Q95R, *S147G*, *Q148R*	T97A, G163R, V151I, L74M
E78004	WT	Q146R, Q95KQ	Q146R, Q95KQ	*Q148R*, *E138K*, *G140GS*, *L74I*	T66I, Q95K, E157Q, S230R	T66A, A128T, *Y143G*, *G163R*, V151I
E78005	WT	R263K	S153Y	S153F	T66A, *Q146I*	T97A, *Y143R*, V151I
E78060	WT	R263K	R263K, M50I	Q146L	T97A Q146R, T66IT	T97A, E157Q, A128AT, V151I
E78110	E157Q	R263K, M50I	R263K	R263K	*T66I*, *R263K*, M50I	*Y143R*
E102430	E157Q	R263K	R263K	R263K, M50I	*E138K*, *Q148K*	V151S, L74LM
E102952	E157Q	S153F	R263K, M50I	R263K	T66I, E92Q	*Q148R*, *E138K*, *G140A*, V151IV
E103211	E157Q	R263K	R263K	R263K, H51N	S147G, *Q148R*, *E138K*	*Y143R*
E103212	E157Q	R263K	R263K	R263K, M50I	H51Y, S147G, T97A	T97A

The underline refers to the de novo aquisition of E157Q during selection

^a^Integrase derived from clinical isolates with or without the E157Q substitution were inserted into integrase-depleted pNL4.3 plasmids. Isolated recombinant viruses were serially passaged in escalating concentrations of dolutegravir (DTG), bictegravir (BIC), cabotegravir (CAB), elvitegravir (EVG) or raltegravir (RAL) over the course of 36 weeks

^b^Genotypic analysis was performed of at weeks 0, 16, 24 and 36–38. The mutations acquired at final week of passage are listed in the order of their first appearance. Mutations highlighted in italics conferred high-level resistance

In contrast, patient-derived pNL4-3 recombinant strains showed a high genetic barrier to resistance for DTG, BIC, and CAB, both in the presence or absence of E157Q. All recombinant strains harbouring patient-derived integrase developed resistance to all INSTIs over 36–38 weeks. With DTG, BIC and CAB, the predominant resistance profile included R263K (n = 11), R263K/M50I (n = 7), and S153Y/F (n = 5).

Selection of strain E78004 with CAB resulted in high level resistance along a Q148R/E138K/G140GS/L74I pathway (Table 4). This isolate developed Q146R/Q95KQ with DTG and BIC, a hitherto unreported profile for both drugs. Resistance profiles associated with escape from EVG drug pressure were associated with T66I and the accumulation of major resistance including Q148R/K, E138K, and S147G. Emergent high-level resistance to RAL were associated with the Y143R/G and Q148R pathways.

### Phenotypic drug susceptibility of CAB- and EVG-resistant viral variants

Phenotypic drug susceptibility to DTG, BIC, CAB, EVG and RAL was deduced in PHA-stimulated CBMC using select clinical isolates (Table 5) and recombinant strains with patient-derived integrase (Table 6).

**Table 5:** Phenotypic drug susceptibility of viral strains to integrase strand transfer inhibitors (INSTIs) harvested at the designated week of selection with cabotegravir (CAB) or elvitegravir (EVG)

Virus selection week drug^a^	Acquired resistance mutations	EC_50_ (nM) in CBMCs (fold-resistance relative to WT control)				
		DTG	BIC	CAB	EVG	RAL
6343 No drug	WT	0.10	0.17	0.23	0.21	ND
6343 Wk 46 CAB	S153Y, G163R	0.60 (6 ×)	1.33 (8 ×)	0.87 (3.2 ×)	0.53 (2.5 ×)	ND
5326 No drug	WT	0.77	0.41	0.20	0.253	1.12
5326 Wk 45 EVG (1 µM)	S263K, S153A	0.52 (0.7 ×)	0.53 (1.3 ×)	0.19 (1.0 ×)	*53* (*212 *×)	1.12 (1.0 ×)
5326 Wk 17 CAB (0.01 µM)	Q148K	1.57 (2.0 ×)	0.90 (2.2 ×)	0.61 (3.1 ×)	*2.96* (*11.8 *×)	3.57 (3.2 ×)
5326 Wk 28 CAB (0.5 µM)	Q148K, G140S, G147GS	3.60 (4.7 ×)	0.72 (1.8 ×)	*8.07* (*40 *×)	*180* (*720 *×)	*66.5* (*60 *×)
5326 Wk 48 CAB (1.0 µM)	Q148K, G140S, S147G, L74M	*125* (*162 *×)	*49.01* (*120 *×)	*139.7* (*700 *×)	*3429* (> *1000 *×)	*1007* (*900 *×)
96USSN20 No drug	WT	0.58	0.77	0.29	0.20	1.60
96USSN20 Wk27 EVG (2.5 µM)	T66I, T97A, Q147G	0.40 (0.7 ×)	0.80 (1 ×)	1.20 (4 ×)	>*100* (> *500 *×)	1.7 (1 ×)
96USSN20 Wk 17 CAB (0.025 µM)	Q148R, E138EK, R263KR	*8.08* (*14 *×)	*5.83* (*8 *×)	*2.40* (*8.3 *×)	*22.35* (*112 *×)	*12.01* (*7 *×)
96USSN20 Wk 27 CAB (0.25 µM)	Q148R, E138K, R263K, L74LM	*10.0* (*17 *×)	*9.01* (*12 *×)	>*30* (> *100 *×)	>*300* (> *1500 *×)	*300* (*188 *×)
96USSN20 Wk 45 CAB (0.5 µM)	Q148R, E138K, R263K, L74M	*13.93* (*24 *×)	*13.40* (*17 *×)	*47.8* (*165 *×)	*1612* (*8060 *×)	*568* (*355 *×)

^a^Viruses were harvested at the designated week of selection, amplified in PHA-stimulated CBMCs and genotyped. Viruses were co-cultured in PHA-stimulated CBMCs to deduce drug susceptibility against dolutegravir (DTG), bictegravir (BIC), cabotegravir (CAB), elvitegravir (EVG) and raltegravir (RAL). Samples in italics represent greater than 5-fold reduction in drug susceptibility

**Table 6:** Phenotypic drug susceptibility of viral strains to integrase strand transfer inhibitors (INSTIs) harvested at the designated week of selection with cabotegravir (CAB) or elvitegravir (EVG)

Viral variant-drug selection week (drug)^a^	Acquired resistance mutations	EC_50_ (nM) in CBMCs (fold-resistance relative to WT control)				
		DTG	BIC	CAB	EVG	RAL
pNL4.3	WT	0.71	0.49	0.39	0.13	–
pNL4.3-R263K	R263K	1.56 (2.2 ×)	1.60 (3.3 ×)	0.91 (2.4 ×)	0.79 (6.1 ×)	–
pNL4.3-S153Y	S153Y	3.34 (4.7 ×)	3.40 (7.0 ×)	1.01 (2.6 ×)	<0.3 (2 ×)	–
pNL4.3-S153F	S153F	0.45 (0.63 ×)	0.63 (1.3 ×)	0.56 (1.4 ×)	<0.3	–
E78004 No drug	WT	0.49	1.26	0.27	0.65	1.18
E78004 Wk18 CAB (0.0025 μM)	Q95KQ, Q148R	1.28 (2.6 ×)	1.17 (0.9 ×)	0.67 (2.5 ×)	*0.90* (*32 *×)	1.64 (1.4 ×)
E78004 Wk26 CAB (0.005 μM)	Q95KQ, Q148R, E138EK	1.60 (3.3 ×)	1.77 (1.4 ×)	*3.04* (*11.3 *×)	*93.57* (*144 *×)	*20.76* (*18 *×)
E78004 Wk36 CAB (0.25 μM)	Q148R, E138K, L74I, G140GS	*12.34* (*25 *×)	*6.08* (*5.3 *×)	*23.6* (*87 *×)	*36.16* (*57 *×)	*3182* (> *100 *×)
E78004 No drug	WT	0.66	0.66	0.43	0.79	1.69
E78004 Wk18 EVG (0.25 μM)	T66I, Q95K, E157EQ	0.59 (0.9 ×)	0.56 (0.9 ×)	0.24 (0.5 ×)	*29.10* (*37 *×)	*14.57* (*8.6 *×)
E78004 Wk26 EVG (0.25 μM)	T66I, Q95K, E157Q	0.53 (0.8 ×)	0.38 (0.6 ×)	0.39 (0.9 ×)	*69.76* (*89 *×)	7.98 (4.7 ×)
E78004 Wk36 EVG (2.5 μM)	T66I, Q95K, E157Q, S230R	0.06 (0.1 ×)	0.01 (0.01 ×)	0.03 (0.1 ×)	*123.10* (*156 *×)	5.47 (3.2 ×)
E78060 No drug	WT	0.52	0.92	0.45	0.46	2.43
E78060 Wk36 RAL (0.5 μM)	T97A, A128AT, E157Q, V151I	0.31 (0.6 ×)	0.30 (0.3 ×)	0.22 (0.5 ×)	*23.79* (*52 *×)	*49.83* (*21 *×)
E102952 No drug	WT (*E157 Q*)	0.09	0.25	0.13	0.33	0.44
E102952 Wk18 RAL (0.025 μM)	Q148R (*E157Q*)	0.17 (1.8 ×)	0.10 (0.4 ×)	0.14 (1 ×)	0.41 (1.3 ×)	0.75 (1.7 ×)
E102952 Wk38 RAL (20 μM)	Q148R, E138K, G140A, V151IV (*E157Q)*	*5.46* (*58 *×)	*2.63* (*10.6 *×)	*2.13* (*16.3 *×)	*1255* (> *100 *×)	*1519* (> *100 *×)

The underline refers to the de novo aquisition of E157Q during selection

^a^Viruses were harvested at the designated week of selection, amplified in PHA-stimulated CBMCs and genotyped. Viruses were co-cultured in PHA-stimulated CBMCs to deduce drug susceptibility against dolutegravir (DTG), bictegravir (BIC), cabotegravir (CAB), elvitegravir (EVG) and raltegravir (RAL). Samples in italics represent greater than 5-fold reduction in drug susceptibility. pNL4.3 recombinant virus are included as controls with R263K and S153Y mutations inserted by site-directed mutagenesis

Here, we showed two isolates 5326 and 96USSN20 serially accumulated resistance mutations with CAB, leading to drug dose escalation of 0.5 and 1 µM, respectively. Viruses were amplified at weeks 8, 16, 24 and 46 weeks (Table 5). The first appearance of Q148K as a solitary mutation under CAB pressure in clinical isolate 5326) and recombinant strain E78004 at weeks 18 conferred low-level (< 2–3 fold) resistance to CAB, DTG, BIC and RAL with moderate (12–32-fold) reduced susceptibility to EVG (Tables 5, 6). For isolate 5326, the progressive accumulation of Q148K/G140S/G147GS resulted in increasingly high cross-resistance to CAB, RAL and EVG while retaining susceptibility to DTG and BIC (Table 3). The resistant variant of 5326 amplified at week 48 under selective CAB pressure, harbouring L74M/G140S/S147G/Q148K mutations showed high-level cross-resistance to all INSTIs, including DTG, BIC, CAB, EVG and RAL (Table 5). Similarly, 96USSN20 and E78004 viruses developed resistance along a Q148R pathway leading to L74M/E138K/G148R/R263K and L74I/E138K/G140S/Q148R conferring cross-resistance to all INSTIs.

Phenotypic drug susceptibility assays explored the potential impact of the E157Q substitution drug susceptibility to INSTIs (Table 6). Viral strains E78004 and E78060 acquiring the E157Q under EVG and RAL showed hypersensitivity to DTG, BIC, CAB, consistent with the observed attenuated development of resistance to RAL and EVG of E157Q relative to wild-type recombinant strains.

One recombinant strain, E78004, acquired a Q148R resistance pathway under selective pressure with CAB. The appearance of Q148R/Q95KQ followed by Q148R/Q95KQ/E138EK resistant strains at weeks 18 and 26 resulted in moderate 2.5- and 11.3-fold resistance to CAB, while retaining susceptibility to DTG and BIC. The outgrowth of the L74I/E138K/G140GS, Q148R showed 25-, 5.3-, 87-, 57- and > 100-fold cross-resistance to DTG, BIC, CAB, EVG, and RAL, respectively.

### Switching EVG-resistant strains to DTG, BIC, or CAB

To gain further understanding of the residual efficacies of DTG, BIC, and CAB on EVG-resistant variants, we performed switch experiments. Six EVG-resistant variants and the pNL4.3 recombinant strain showed high-level resistance at week 46, growing in the presence of 1–2.5 µM EVG. These resistant variants were amplified at week 47 and switched to serial drug-dose escalations with DTG, BIC or CAB for a further 27 weeks.

As summarized in Table 7, The EVG-resistant variants retained residual susceptibility to second generation INSTIs. There was however, broader antiviral sensitivity to DTG and BIC than CAB. Drug dose escalations with the latter three drugs were initiated at 0.001 µM. Following passage for 17 weeks, drug-dose escalations reached 0.023 ± 0.012 µM, 0.027 ± 0.006 µM, and 0.121 ± 0.034 at week 27. Drug dose escalations were significantly higher for CAB than DTG or BIC (Bartlett’s statistic = 14.16, p = 0.0008, p < 0.05, post hoc Tukey’s test).

**Table 7:** Viral outgrowth of elvitegravir (EVG) resistant viruses switched to dolutegravir (DTG), bictegravir (BIC) or cabotegravir (CAB)

Virus	Initial EVG selection	Drug switch selection		Resistance mutations at week 27	Lost EVG mutations	Acquired mutations
		2nd drug	Drug (µM)			
6343	EVG Wk 46	Pre-switch	0.25	T66I, R263K		
6343		DTG	0.010	M50MI, R263K	T66I	M50IM
6343		BIC	0.050	R263K	T66I	
6343		CAB	0.050	R263K	T66I	
6343		No drug		R263K	T66I	
14637	EVG Wk 46	Pre-switch	1.0	T66I, E157Q, R263K		
14637		DTG	0.010	E157Q, R263K	T66I	
14637		BIC	0.025	E157Q, R263K	T66I	
14637		CAB	0.050	E157Q, R263K	T66I	
14637		No drug		E157Q, R263K	T66I	
5326	EVG Wk 46	Pre-switch	1	S153A, R263K		
5326		DTG	0.010	S153A, R263K		
5326		BIC	0.010	S153A, R263K		
5326		CAB	0.050	S153A, R263K		
5326		No drug		S153A	R263K	
14624	EVG Wk 46	Pre-switch	1	T66I		
14624		DTG	0.005	T66I, L74M, E138K, S147G, M154IM		L74M, E138K, S147G, M154IM
14624		BIC	0.010	H51HY	T66I	H51HY
14624		CAB	0.10	T66I, L74M, G140GS, S147GS		L74M, G140GS, S147GS
14624		No drug		T66I		
14947	EVG Wk 46	Pre-switch	5	T66I, E138EK, S147G, Q148R		
14947		DTG	0.005	T66I, E138EK, S147G, Q148R, S230N		S230N
14947		BIC	0.025	T66I, E138EK, S147G, Q148R, S230N		S230N
14947		CAB	0.100	T66I, L74M, E138EK, S147G, Q148R, S230N		L74M, S230N
14947		No drug		T66I, S147G, S230NS	E138EK, Q148R	S230NS
96USSN20	EVG Wk 46	Pre-switch	2.5	T66I, Q146R, S147G		
96USSN20		DTG	0.100	T66I, Q146R, S147G, E138E1AEKT, Q148R	Q146R	E138AEKT, Q148R
96USSN20		BIC	0.050	T66I, Q146R, S147G, E138A, T97A	Q146R	T97A, E138A, Q148R
96USSN20		CAB	0.25	T66I, Q146QR, S147G, E138A, Q148QR	Q146QR	E138A, Q148QR
96USSN20		No drug		T66I, S147G	Q146R	
pNL4.3	EVG Wk 46	Pre-switch	2.5	T66I, T97A, S147G, V151I, S153A		
pNL4.3		DTG	0.025	T66I, T97A, S147G, V151I, S153A		
pNL4.3		BIC	0.025	T66I, T97A, S147G, V151I, S153A		
pNL4.3		CAB	0.250	T66I, T97A, S147G, V151I, S153A		
pNL4.3		No drug		T66I, T97A, S147G, V151I, S153A		

Patient-derived viral strains, subjected to EVG selective pressure for 46 weeks, were switched to serially increasing concentrations of DTG, BIC, CAB or no drug for 27 weeks. Genotyping was performed at week 27 and re-genotyped to monitor the loss and acquisition of mutations

Genotypic analysis showed the switch of EVG-resistant strains (n = 6) to DTG, BIC, CAB or a no drug control for 27 weeks resulted in the loss of the T66I (3/6 selections) or Q146R (1/6) substitutions associated with primary resistance to EVG (Table 7). For isolate 6343, the loss of T66I was accompanied by the acquisition of M50I with DTG (Table 7). The EVG-resistant isolates 6343, 14637 and 5326, harbouring R263K, E157Q/R263K and S153A/R263K, showed residual susceptibility to DTG, BIC and CAB with no further acquisition of resistance mutations at week 27 (Table 7).

The EVG-resistant 14624 T66I variant displayed a higher residual antiviral susceptibility to BIC than DTG and CAB (Table 7). With BIC, T66I was lost and H51HY was acquired. In contrast, the loss of T66I in 14624 was accompanied by the acquisition of L74M/E138K/S147G/M154IM and L74M/G140GS/S147G at week 27 with DTG and CAB, respectively.

The EVG-resistant 14947 variant T66I/E138K/S147G/Q148R variant accumulated S230N with BIC and DTG and L74M/S230N with CAB. The 96USSN20 virus resistant to EVG (T66I, Q146R, and S147G) lost Q146R and acquired E138A and Q148R with DTG, BIC and CAB. The final concentrations at week 27 revealed CAB escape.

Taken together, DTG, BIC and CAB showed broad antiviral efficacies against wild-type and EVG-resistant viruses. BIC and DTG appear to be better able to inhibit viral replication than CAB in several primary HIV-1 isolates and EVG-resistant variants.

## Discussion

The findings in this study demonstrated that DTG and BIC showed higher barriers to resistance than EVG. With EVG, resistance was observed in all 12 clinical isolates. In 4/12 and 6/12 selections with DTG and BIC, no resistance mutations arose in long-term passage, respectively. In the remaining selections, the acquisition of singleton R263K, S153Y/F or H51Y substitutions conferred low-level resistance, regardless of viral subtype. Although 4/12 and 6/12 selections with CAB, yielded no resistance or minor resistance, respectively, two selections with CAB (one subtype B and one CRF02_AG), resulted in the acquisition of Q148R/K with multiple secondary resistance substitutions conferring high-level cross-resistance to all INSTIs.

Although there is a high correlation in the genotypic and phenotypic characteristics associated with resistance to DTG and BIC, CAB may show a lower barrier to resistance. In selections of two clinical isolates and a patient-derived recombinant strain, resistance to CAB arose through a Q148R. The first appearance of Q148R showed < 3-fold resistance to CAB, DTG and BIC. The sequential accumulation of mutations by these three strains resulted in Q148R/E138K/R263K/L74M, Q148K/G140S/S147G/L74M and Q148R/E138K/L74I/G140GS mutational motifs conferring in high-level cross-resistance to all five INSTIs.

BIC and CAB, like DTG displayed antiviral efficacies against viral variants acquiring EVG-resistance mutations. Overall, BIC and DTG were superior to CAB. This is consistent with recent studies modelling the respective binding of CAB, BIC and DTG within the active site of the integrase enzyme [31].

The selection of Q148R in CAB selections is consistent with the observed acquisition of Q148R in two patients in the Latte clinical trials [14, 32]. Molecular models suggest that there is more conformational rigidity with CAB than DTG in metal-chelating scaffold leading to potential steric interactions between CAB and the Q148 locus [41]. The potential steric interactions induced by Q148R at and near the β4–α2 loop may affect binding kinetics of CAB, leading to a decreased dissociative half-life with Q148R between that of DTG and RAL [41].

CAB, an analogue of DTG, has been formulated as an oral tablet (half-life 40 h) and as a long-acting injectable nanosuspension with an intramuscular and subcutaneous half-life of 40 days. CAB/RIL has shown durable viral suppression in patients who are suppressed to less than 50 copies/ml, providing proof of principle for its’ use in two-drug maintenance therapy, as well as a potential PreP strategy [14, 32, 42]. The observed emergence of resistance through the Q148R pathway may lead to cross-resistance to the entire class of INSTIs. Although in vitro findings may not arise in the clinic, careful attention may be needed to assure drug adherence and prevent tail periods of declining drug in injectable formulations.

It is noteworthy that HIV-1 viral variants may differ in their replicative fitness and their ability to override resistance bottlenecks. In our previous studies, we showed that viral variants associated with large cluster transmission outbreaks may show a facilitated development of resistance to DTG and EVG than viruses leading to singleton transmission [25]. In this study, isolates 14637 and 14947 were associated with large cluster outbreaks.

This study utilized a panel of clinical isolates reflective of newly-infected treatment-naïve persons harbouring CCR5 viruses. Our analysis also included recombinant CXCR4-tropic pNL4.3 recombinants constructs encoding patient-derived integrase. Most reported studies to date, have limited their performed in vitro analysis of BIC and CAB using pNL4.3 vector constructs or MT-2 or MT-4 cells [31, 43–45].

In previous studies, subtype C variant, 4742, developed the G118R mutation in cell culture selections with DTG and Merck investigation INSTI, MK2048 [46]. The development of G118R was associated with a rare GGA natural polymorphism at codon 118, facilitating a G to A transition leading to G118R (AGA). In this study, the 4742-viral strain gave rise to no resistance with either DTG or BIC, R263KR with CAB, and E92EG/R263K with EVG.

Taken together, our findings show improved resistance profiles for DTG and BIC in all tested viral strains. The CAB, an analogue of DTG with high potency, may be prone to the development of Q148K/R leading to cross-resistance to the entire class of integrase inhibitors. The high potency of DTG and BIC confirms their suitability for use in resource-limited settings dominated by non-B subtypes. A larger panel of viral isolates are needed to address the potential development of the Q148R/K resistance pathway with CAB [43].

## Conclusions

The advent of integrase inhibitors has transformed the management of HIV-1 infection. Although treatment failure with RAL and EVG can result in the emergence of INSTI resistance, DTG and BIC have been quite impervious to the development of resistance in the clinical setting [15, 47, 48]. To date, there have been few reports of virological failure and resistance in treatment-naïve persons receiving triple combination DTG-containing regimens [47, 48] and in virologically suppressed patients receiving DTG monotherapy [46, 49]. The present study used in vitro selections using viral isolates from 20 persons to show patterns of resistance to DTG, BIC and CAB that may potentially arise in real-world settings. Our findings indicate that drug resistance monitoring in patients on INSTI-based regiments is essential, despite the high-genetic barriers of these drugs. Although in vitro experiments might not always reflect what, will happen in patients, resistance may be breached when INSTIs are given in monotherapy and in select patients failing INSTI-based regimens [50]. As treatment options coalesce around the use of second-generation integrase inhibitors in resource-limited settings, more information is needed on emergent resistance to DTG, BIC and CAB and their potential impact on viral response to later-line treatment options [50].

## Methods

### Cells and antiviral compounds

BIC and EVG were kindly provided by Gilead Sciences Inc. (Foster City, California). CAB was purchased from Toronto Research Chemicals (Toronto, Canada). DTG was kindly provided by ViiV Healthcare (Research Triangle Park, Inc). MT-2 and 96USSN20 cells were obtained from the NIH AIDS Reagent program, Division of AIDS, NAID, NIH with cell line provided by Dr D Richman and Drs D Ellenberger, P Sullivan and RB Lai, respectively [51, 52]. Cord blood mononuclear cells (CBMCs) were isolated as previously described from non-nominative discarded blood obtained through the Department of Obstetrics, Jewish General Hospital [53]. The CEM-GXR cells and HIV-1 pNL4.3 delta integrase plasmid (Δint) were kindly provided by Dr. Mark Brockman (Simon Fraser University, Burnaby, Canada [54].

### Isolation of patient-derived HIV-1 primary isolates and recombinant strains encoding patient-derived HIV-1 integrase

The FRQS-Réseau SIDA supports a representative cohort of newly-infected persons with clinical indication of primary infection. In this study, HIV-1 strains were isolated from seven subjects harboring subtype B infections and four subjects harboring non-B subtype infections. HIV-1 isolates were amplified as previously described through co-culture of patient CD8-depleted peripheral blood mononuclear cells (PBMCs) with CD8-depleted phytohemagglutinin-stimulated CBMCs [53, 55]. Amplified cell-free viral supernatants were tittered and stored at − 70 °C until use [53, 55]. In addition, viruses were amplified from the 96USSN20 strain (subtype CRF02_AG) and the pNL4.3 subtype B reference clone obtained from the NIH AIDS Reagent program, Division of AIDS, NIAID, NIH.

Clinical plasma samples were also obtained from treatment-naive persons harboring the WT (n = 5) or E157Q (n = 5) substitution in integrase. Patient-derived amplicons encoding the integrase gene (1064 nucleotides long) were inserted into the pNL4-3 recombinant vector as previously described [54]. Briefly, HIV-1 was RT-PCR amplified from plasma HIV RNA using sequence-specific subtype B primers [56, 57]. Second round PCR was performed using Expand™ High Fidelity Enzyme (Roche Diagnostics, Laval, Quebec) with forward (IN4155F-5′-GTACCAGCACACAAAGGAATTGGAG) and reverse primer (IN5219R-5′-CCTAGTGGGATGTGTACTTCTGAAC). primers. Recombinant viruses were generated by co-transfecting sequence verified second-round PCR amplicons with linearized Δint-pNL4.3 into CEM-GXR cells via electroporation cells [58]. Transfection cultures were maintained and resulting viruses harvested as described previously [54]. Upon harvest, recombinant viruses were sequence validated.

Amplified infectious clinical isolates and recombinant viral stocks were genotyped and stored at − 70 °C. GenBank accession numbers and integrase polymorphisms are indicated in Tables 1 and 2.

### Cell culture-based selection of resistance to integrase inhibitors

Selections of HIV-1 variants resistant to INSTIs were performed through serial passage of patient-derived clinical isolates (n = 12) or recombinant strains (n = 10) in CBMCs, in the presence of serially escalating concentrations of DTG, BIC, CAB and EVG over the course of 36–46 weeks, as previously described [53, 59–61]. At each passage, aliquots of cell-free supernatant were stored at − 70 °C for further analysis.

Stepwise drug-dose escalations were based on weekly reverse transcriptase (RT) enzymatic assays performed for each isolate in the presence and absence of the relevant INSTI [34]. Briefly, 10 µl clarified culture supernatant were incubated in a 50 µl reaction mixture containing 50 mM Tris (pH 8.0), 5 mM MgCl_2_, 150 mM KCl, 5 mM dithiothreitol, 0.3 mM glutathione, 0.5 mM EGTA, 0.05% Triton X-100, 50 µg of poly(rA)–oligo(dT)_12–18_ per ml and 0.1 µCi of [^3^H-TPP]. After a 4 h incubation at 37 °C, 150 µl of ice-cold 10% trichloroacetic acid (TCA) was added for 30 min at 4 °C to precipitate incorporated [3H]-TTP. The precipitated mixture is transferred onto a Millipore multiscreen Glass Fiber FC plates with 10% TCA, vacuum drained. and washed twice with 10% TCA and once with cold ethanol using a Millipore multiscreen manifold. Scintillation cocktail (30 µl) is added to each well. Radioactivity is measured using a Perkin Elmer MicroBeta Trilux microplate counter.

Residual viral efficacy to DTG, BIC and CAB against EVG-resistant viruses was assessed by switch experiments. EVG-resistant viruses isolated at week 46 were grown in escalating concentrations of DTG, BIC, CAB or no drug control for a further 17 and 27 weeks. Genotyping at week 17 and 27 monitored the loss of EVG-associated resistance mutations and acquisition of mutations to second generation INSTIs.

Genotypic analyses were performed at weeks, 8, 16, 27–30 and 46 weeks to evaluate the acquisition and accumulation of amino acid substitutions that could be associated with reduced susceptibility to antiretroviral drugs (i.e., drug resistance mutations). Sanger (population) sequencing of viral RNA extracted from culture supernatants across the integrase coding regions was performed as previously described [60, 62].

Phenotypic susceptibility to DTG, BIC, CAB, EVG and RAL were monitored using a cell-based in vitro assay. Briefly, viruses were amplified from stored cell culture supernatants at designated weeks following in vitro selection. Resistant and wild-type control viruses were infected with serial dilutions of INSTIs. After 7 days, culture supernatants were collected and analyzed for RT activity. The 50% effective concentrations were calculated on the analysis of dose–response curves using GraphPad Prism version 6.07 software.
